# Recent Advances in the Synthesis of Isoquinoline-Fused Benzimidazoles

**DOI:** 10.3390/molecules27072062

**Published:** 2022-03-23

**Authors:** Dylan J. Twardy, Kraig A. Wheeler, Béla Török, Roman Dembinski

**Affiliations:** 1 Department of Chemistry, Oakland University, 146 Library Dr., Rochester, MI 48309, USA; djtwardy@oakland.edu; 2 Department of Chemistry, Whitworth University, 300 W. Hawthorne Rd., Spokane, WA 99251, USA; 3 Department of Chemistry, University of Massachusetts Boston, 100 Morrissey Blvd., Boston, MA 02125, USA; 4 Centre of Molecular and Macromolecular Studies, Polish Academy of Sciences, Sienkiewicza 112, 90-363 Łódź, Poland

**Keywords:** benzo[4,5]imidazo[2,1-*a*]isoquinoline, benzimidazo[2,1-*a*]isoquinoline, benzimidazole, isoquinoline, nitrogen heterocycles

## Abstract

This review includes recent developments in the synthesis of benzo[4,5]imidazo[2,1-*a*]isoquinolines with particular attention given to categorizing protocols based on the structural features of the ring architecture and crystallographically characterized reaction products.

## 1. Introduction

**Figure 1:**
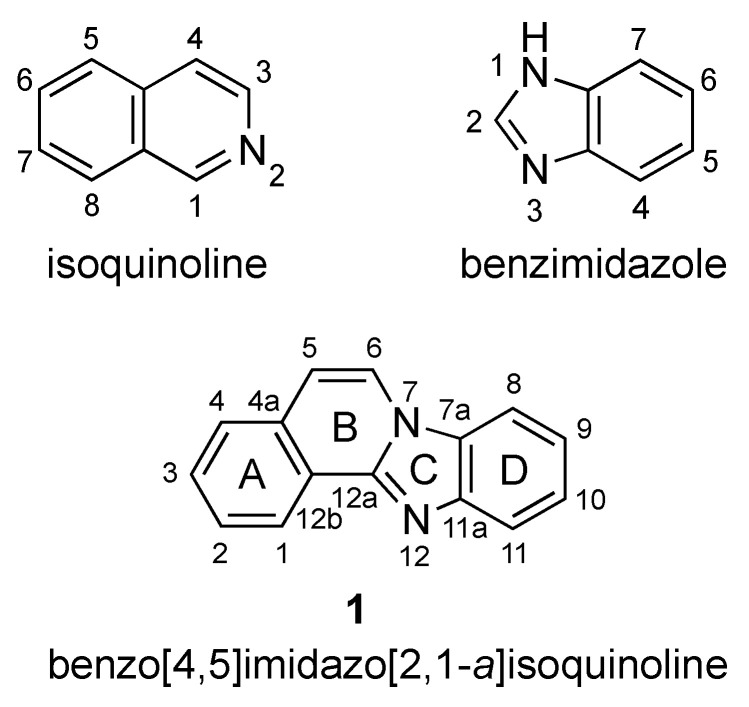
Isoquinoline, benzimidazole, and benzimidazo[2,1-*a*]isoquinoline **1**.

Benzo[4,5]imidazo[2,1-*a*]isoquinoline **1** is a heterotetracyclic compound that combines fused benzimidazole and isoquinoline moieties (Figure 1), with each structural feature exhibiting broad and multifaceted biological activity. The association of these two ring systems has so far resulted in studies directed at the inhibition of topoisomerase I [1] and the cyclic AMP-dependent protein kinase (PKA) catalytic subunit [2], as well as anticancer activities [3].

Synthetic protocols for this class of compounds often include the use of metal reagents/catalysts. Separation of the targeted organic product(s) from these metal-containing catalysts or reagents is required to minimize sources of auxiliary toxicity and meet the standards of biological testing or specific pharmaceutical thresholds [4]. This review highlights the development of methods that reduce cost, improve product selectivity or purity, and offer more effective routes for the synthesis of benzimidazo[2,1-*a*]isoquinolines. Moreover, emphasis is placed on green methods that support contemporary environmental and safety improvements.

The seminal work of Sun and LaVoie (1996) provided one of the earliest comprehensive synthetic routes to benzimidazo[2,1-*a*]isoquinolines [1]. Since then, especially within the years of 2016–2021, researchers have developed and refined a variety of alternative synthetic protocols for these molecules. Figure 1 provides a labeling scheme for the rings of benzimidazo[2,1-*a*]isoquinolines to facilitate the sorting of synthetic methods as it relates to specific ring formation. Within each group of ring(s) formation, the reactions based on traditional halogen-containing components are presented first, followed by C–H activation-based protocols. Beyond the four-ring system, selected references that describe homologs containing extended fused aromatic systems are occasionally included.

## 2. Ring B Formation

Aryl-substituted benzimidazoles are versatile starting materials that necessitate the construction of a C–5/C–6 vinylene bridge between the *ortho* position of an aryl group (C–4a in ring A) and the benzimidazole unit (N–7 in ring C, Figure 1). The source of the -CH=CH- moiety may vary. The functionalization of an aryl ring via a halogen atom provides a convenient anchor for such a synthetic approach.

**Figure 2:**
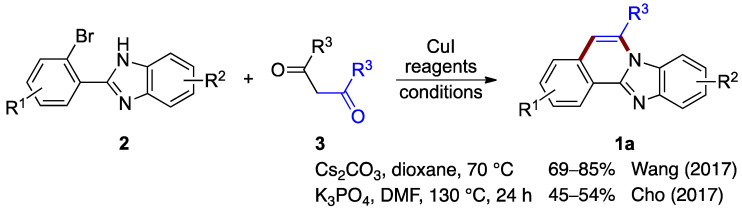
Copper(I)-catalyzed coupling protocols with *o*-bromophenyl benzimidazoles **2** and 1,3-diketones **3** [5,6].

Wang et al. [5] and Cho et al. [6] independently reported protocols for forming benzimidazo[2,1-*a*]isoquinolines by reacting *o*-bromophenyl-substituted benzimidazoles **2** with substituted 1,3-diketones (**3**; Figure 2). Both procedures proceed by copper-catalyzed coupling followed by a deacylation (debenzoylation) step. Wang et al. optimized reaction yields for the 2-(2-bromo-phenyl)-1*H*-benzo[*d*]imidazole **2** and the diphenyl 1,3-diketone (dibenzoylmethane) **3** using copper(I) iodide as the catalyst in the presence of cesium carbonate (69–85% yield) [5]. Cho et al. optimized a similar copper-catalyzed reaction using potassium phosphate as a base (45–54% yield) [6]. These protocols show the utility of various diphenyl 1,3-diketones for the production of benzimidazo[2,1-*a*]isoquinolines **1a** (Figure 2).

**Figure 3:**
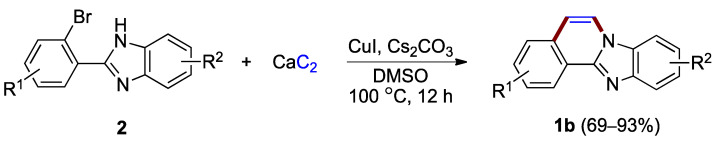
Copper(I)-catalyzed coupling protocol using *o*-bromophenyl benzimidazoles **2** and calcium carbide as reactants.

More recently, Liu and Li reported the application of calcium acetylide (calcium carbide), a low-cost industrial commodity, for the synthesis of benzimidazo[2,1-*a*]isoquinolines from *o*-haloaryl-substituted benzimidazoles **2** (Figure 3) [7]. The protocol was used to prepare a variety of benzimidazo[2,1-*a*]isoquinoline derivatives **1b** with no substituents at the C–5 and C–6 positions (69–93%), while tolerating halogens such as chlorine and fluorine attached to the phenyl ring.

**Figure 4:**
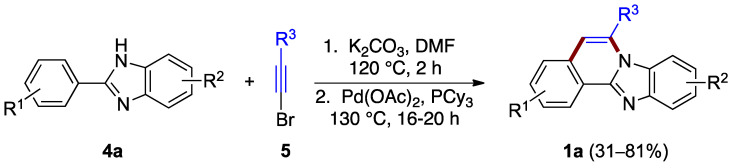
Hydroamination of bromoacetylene **5** by benzimidazole **4a**.

The ring B formation can be achieved with the halogen located on the alkyne reagent as well. Li et al. reported a two-step synthesis that uses phenyl benzimidazole **4a** and bromoacetylene **5** derivatives (Figure 4) [8]. The first step is thought to involve nucleophilic addition (hydroamination) of the benzimidazole to bromoalkyne **5** followed by palladium-catalyzed C–H vinylation. This process effectively generates a bond between the benzimidazole phenyl substituent leading to ring B formation and the benzimidazo[2,1-*a*]isoquinoline **1a** product (31–81% yield). The ortho regioselectivity results from the length of already anchored bromovinyl intermediate (Figure 4).

**Figure 5:**
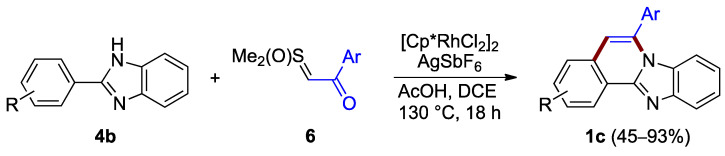
Annulation of benzimidazole **4b** with sulfoxonium ylides **6**.

Ring B can also be formed in the absence of a halogen anchor. Cheng et al. reported the synthesis of benzimidazo[2,1-*a*]isoquinolines **1c** through the annulation of non-halogenated 2-arylimidazoles **4b** and the acetophenone-derived sulfoxonium ylide **6**, under aerobic conditions (45–93% yield; Figure 5) [9]. The procedure relies on a rhodium species generated in situ, facilitating a C–C coupling and forming a C–N bond. The dichloro(pentamethylcyclopentadienyl)rhodium(III) precatalyst plays an important role in the reaction process where it is activated by acetic acid combined with a catalytic amount of silver hexafluoroantimonate.

**Figure 6:**
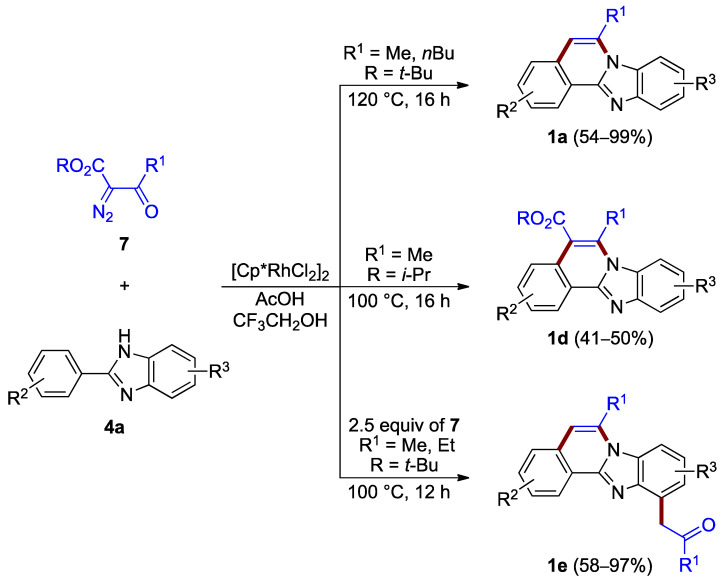
Synthesis of functionalized benzimidazo[2,1-*a*]isoquinolines **1a**,**d**,**e** via rhodium(III)-catalyzed annulation of α-diazoketoesters **7** and 2-arylbenzimidazoles **4a**.

During the same time period, Song et al. reported the synthesis of benzimidazo[2,1-*a*]isoquinolines **1a** (54–99%) and **1d** (41–50%) via the rhodium(III)-catalyzed [4 + 2] annulation of α-diazoketoesters **7** and 2-arylbenzimidazoles **4a**. The choice of appropriate ester groups (–COO-*t*-Bu or –COO-*i*-Pr) or additive (AcOH) controls the reaction outcome with up to two C–C bond-breaking steps (retro-Claisen and decarboxylation) during the C–H functionalization/condensation process (Figure 6 top and center) [10]. The related reaction also produced 5-hydroxy C5–C6 saturated derivatives (not illustrated).

The presence of excess of diazo compounds **7**, allows for double C–H activation and the addition of a methylene ketone unit, and leads to more functionalized derivatives **1e** (58–97%; Figure 6 bottom) [11].

**Figure 7:**
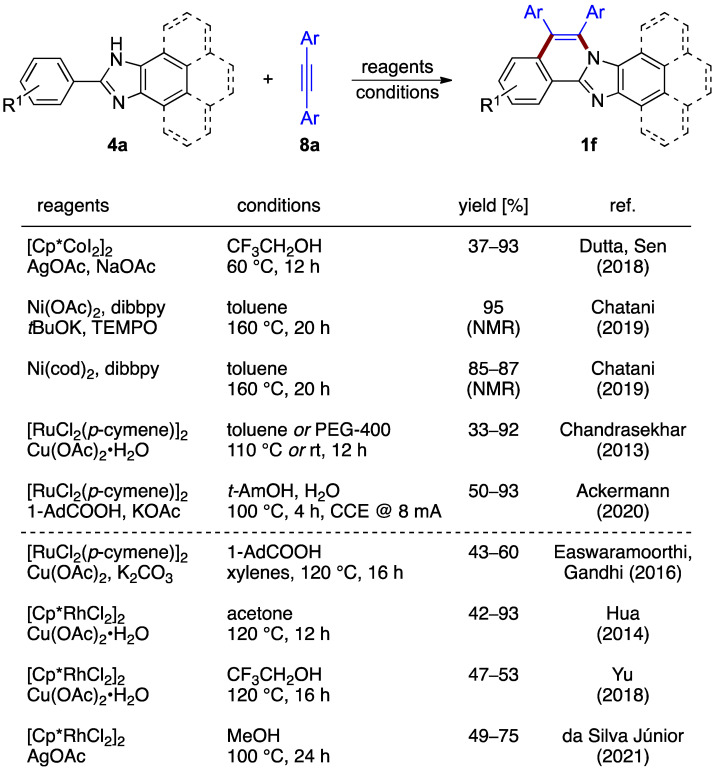
Oxidative annulation of 2-phenylbenzimidazoles **4a** and their fused homologs with internal alkynes **8** by different protocols [12,13,14,15,16,17,18,19].

The oxidative annulation procedures rely on the formation of a vinylene unit between C–H and N–H moieties. The non-halogenated 2-arylbenzimidazoles **4a** and a variety of diarylacetylenes **8a** react in the presence of a catalytic amount of cobalt(III) catalyst and silver acetate as an oxidant, as reported by Dutta and Sen, yielding structures **1f** (37–93%; Figure 7) [12]. Nickel complexes also have been shown by Chatani et al. to be active for an analogous reaction with diphenyl acetylene **8a** (Ar = Ph) [13]. Various protocols featuring the presence or absence of base were elaborated (Figure 7). A similar synthesis catalyzed by [RuCl_2_(*p*-cymene)]_2_ was also reported (Chandrasekhar et al.; 43–60% yield) [14]. Another ruthenium-catalyzed process, elaborated by Ackermann et al., integrated an innovative electrochemical oxidation approach [15]. Molecular hydrogen is the sole byproduct, with the reaction proceeding with 50–93% yields (Figure 7). The key catalytic intermediate, aza-ruthena(II)-bicyclo-[3.2.0]-heptadiene, was isolated and characterized, increasing the mechanistic understanding of the process.

The use of alkynes in the reaction design strategy was applied to the synthesis of related phenantroimidazolo- and pyrenoimidazolo-derived compounds. In these cases, the reaction outcomes were achieved by [RuCl_2_(*p*-cymene)]_2_ (Easwaramoorthi and Gandhi et al.; 43–60% yield) [16] or [Cp*RhCl_2_]_2_ (Hua et al.; 42–93% yield) [17], catalysts aided by copper(II) acetate as the oxidant. A related multicomponent protocol using a [Cp*RhCl_2_]_2_ catalyst for the synthesis of phenantroimidazolo structures was described by Yu et al. [18]. The use of silver acetate oxidant combined with a [Cp*RhCl_2_]_2_ catalyst was reported as well (da Silva Júnior et al.; 49–75% yield) [19].

**Figure 8:**
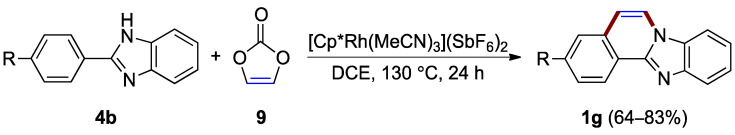
Application of vinylene carbonate **9** in rhodium-catalyzed oxidative annulation of 2-arylbenzimidazoles **1g**.

Without a demand for C–5 and C–6 substituents, vinylene carbonate **9** offers an effective source of the C–4a to N–7 bridging unit in the rhodium(III)-catalyzed annulation of 2-arylbenzimidazoles **4b**, as reported by Nishii, Miura et al. (Figure 8) [20]. This protocol eliminated the need for an additive since vinylene carbonate acts as an internal oxidant to regenerate the rhodium(III) species while producing benzimidazo[2,1-*a*]isoquinolines **1g** (64–83% yield).

**Figure 9:**
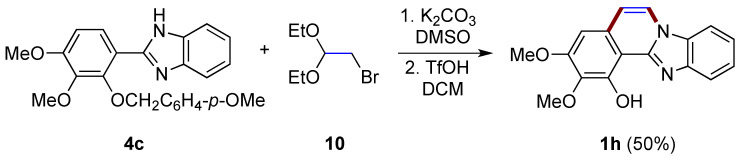
Synthesis of diversely functionalized benzimidazo[2,1-*a*]isoquinoline **1h** with the use of bromoacetaldehyde derivative **10**.

A two-step synthesis first involving the *N*-alkylation of 2-arylbenzimidazole **4c** with bromoacetaldehyde diethyl acetal **10** and then the triflic acid-catalyzed intramolecular cyclization/deprotection of the phenolic hydroxy group allows the formation of benzimidazo[2,1-*a*]isoquinoline **1h** in a moderate yield (50%; Figure 9), as described by Takagi et al. [21]. As clarified by the authors, this same reaction cannot be achieved using concentrated HCl.

**Figure 10:**
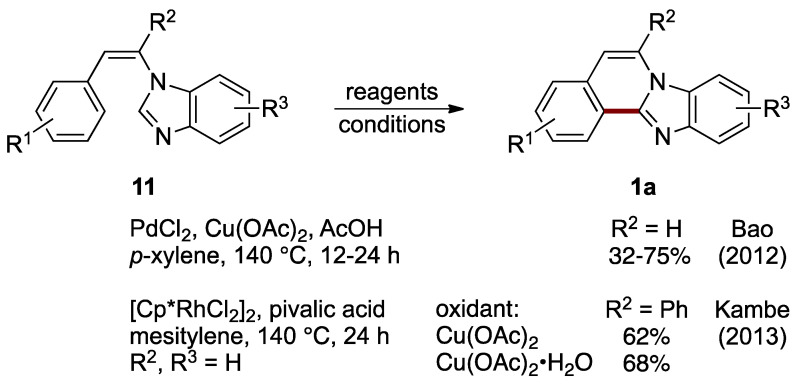
Palladium- and rhodium-catalyzed intramolecular cross-coupling of *N*-styrylbenzimidazoles **11** [22,23].

Formation of ring B can be accomplished by connecting C–12a and C–12b atoms (see Figure 1 for the numbering of structure **1**). Bao et al. achieved oxidative intramolecular C–C bond formation via double sp^2^ C–H activation between the 2-position of an imidazole ring and a benzene ring of *N*-styrylbenzimidazoles **11** catalyzed by palladium(II) chloride, with copper(II) acetate as an oxidant and acetic acid as an additive. This route effectively leads to C–5 and C–6 unsubstituted benzimidazo[2,1-*a*]isoquinolines **1a** (R^2^ = H, 32–75%; Figure 10) [22]. Soon after that report, Kambe et al. described a related oxidative cross-coupling with the aid of a dichloro(pentamethylcyclopentadienyl)rhodium(III) catalyst [23]. The protocol uses copper(II) acetate as an oxidant and pivalic acid as an additive to yield 6-phenylbenzimidazo[2,1-*a*]isoquinolines **1a** (62–68%). The optimized conditions were applied to a phenyl-substituted *N*-styrylbenzimidazole **11** (R^2^ = Ph) to compare anhydrous and monohydrate copper(II) acetate oxidation reagents (Figure 10).

## 3. Ring C Formation

**Figure 11:**
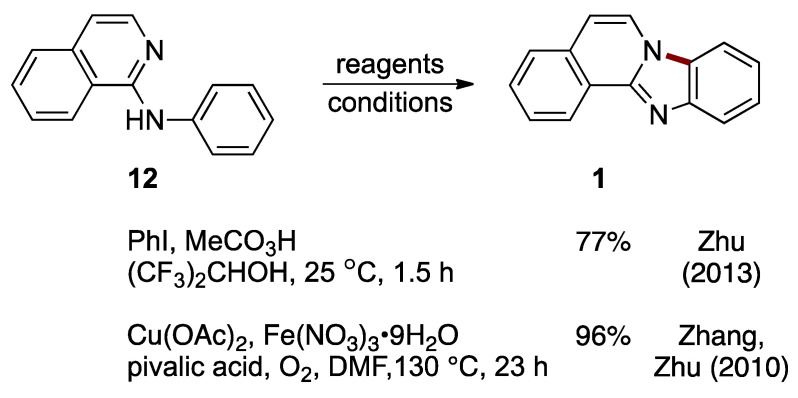
C–H cycloamination of *N*-phenyl-2-aminoisoquinoline **12** catalyzed by an in situ-generated hypervalent iodine reagent or copper/iron bimetallic system [24,25].

A metal-free synthesis of benzimidazo[2,1-*a*]isoquinoline **1** from *N*-phenyl-1-aminoisoquinoline **12** was developed by Zhu et al. This C–H cycloamination reaction is catalyzed by hypervalent iodine(III) generated in situ from iodobenzene (catalytic) and peracetic acid (stoichiometric). The reaction proceeded under ambient conditions with a 77% yield (Figure 11) [24]. Earlier work by Zhang, Zhu et al. established the preparation of benzimidazo[2,1-*a*]isoquinoline **1** (96%) using direct intramolecular aromatic C–H amination of *N*-phenyl-1-aminoisoquinoline **12**, catalyzed by copper(II) acetate and iron(III) nitrate nonahydrate (Figure 11). In this process, the pyridinyl nitrogen in the starting material **12** acts as both a directing group and nucleophile [25].

## 4. Ring B and C Formation

**Figure 12:**
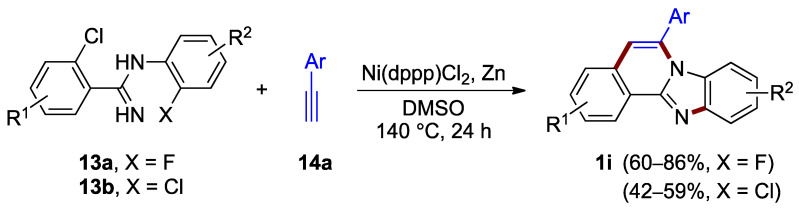
Nickel-catalyzed annulation of chlorohalobenzimidamides **13** with terminal alkynes **14a**.

A method that facilitates the construction of both the imidazole ring C and the pyridine ring B utilizes two halogen anchors. Nickel complexes offer an effective tool for activating the reaction of these halogens and terminal alkynes. Xiao, Deng et al. reported the formation of benzimidazo[2,1-*a*]isoquinoline **1i** as a major product in the nickel-catalyzed annulation of 2-chloro-*N*-(2-halophenyl)benzimidamides **13** with terminal alkynes such as **14a**, under anhydrous conditions and elevated temperature (140 °C; Figure 12) [26]. The yield of the product is dependent on halogen in the starting material. The inspection of the yields for benzimidazo[2,1-*a*]isoquinolines **1i** revealed the increased reactivity of the fluoroaryl reactant **13a** (X = F; 60–86% yield) as compared to the chloroaryl reactant **13b** (X = Cl; 42–59% yield).

**Figure 13:**
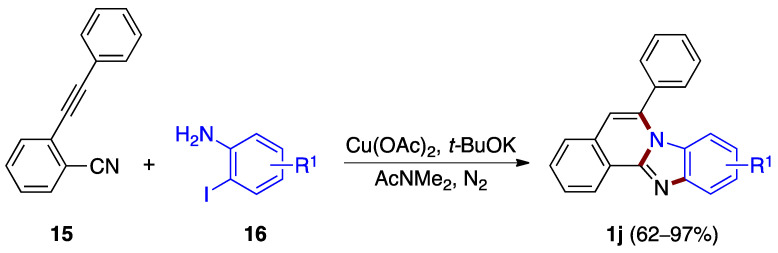
Copper-catalyzed annulation between *o*-alkynylbenzonitrile **15** and *o*-iodoanilines **16**.

A copper(II) acetate-catalyzed cascade cyclization reaction between *o*-alkynylbenzonitrile **15** and *o*-iodoanilines **16** was reported by Deng, Liang et al. [27]. The reaction allows the synthesis of benzimidazo[2,1-*a*]isoquinolines **1j** in good to excellent yields (62–97%), featuring the sequential formation of three different C–N bonds in the presence of a base (Figure 13).

**Figure 14:**
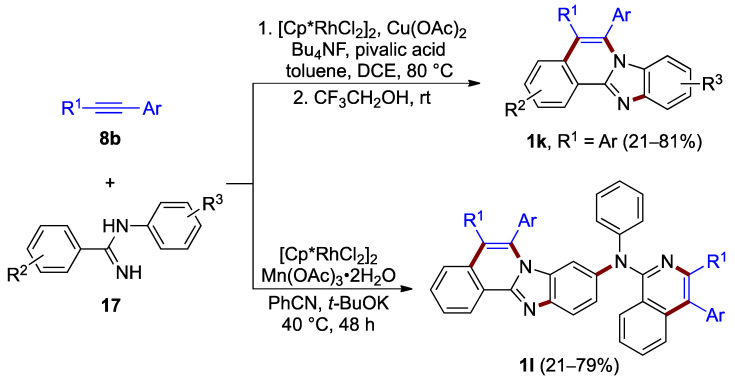
Rhodium-catalyzed annulation (and amination, bottom) of amidines **17** with alkynes **8b**.

The rhodium(III)-catalyzed oxidative annulation of *N*-(2-phenyl)benzimidamide **17**, which proceeds with use of aryl acetylene **8b** at 80 °C, was elaborated by Xu et al. (21–81% yield; Figure 14, top) [28]. This double C–H activation process proceeds by a one-pot reaction, presumably with C–H bond cleavage occurring preferentially at the *N*-phenyl ring. The resulting benzimidazo[2,1-*a*]isoquinoline **1k** features identical aryl substituents at C–5 and C–6 (R^1^ = Ar). Xu, Liu, Du et al. achieved a more architecturally complex structure using alternative reagents and solvents, including manganese(III) acetate as an oxidant instead of copper(II) acetate. The synthetic path for this process comprises multiple C–H activation, an intermolecular meta-selective C–H amination, an intramolecular C–H amination between amidines **17** and alkynes **8b**, and two different Rh(I)–Rh(III) catalytic cycles to yield the 9-arylamino-substituted product **1l** (21–79% yield; Figure 14, bottom) [29].

## 5. Ring B and C Formation Using *o*-Phenylenediamines

**Figure 15:**
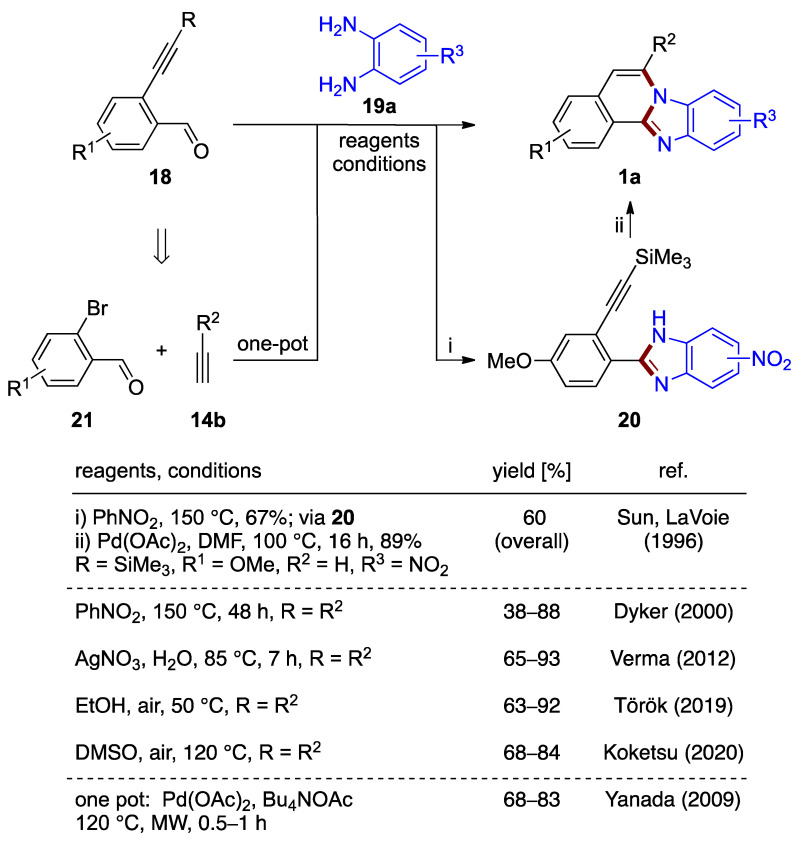
Synthesis of benzimidazo[2,1-*a*]isoquinoline **1a** by reaction of 2-ethynylbenzaldehyde **18** and *o*-phenylenediamine **19a** (top) and by a one-pot reaction of bromobenzaldehydes **21**, terminal acetylenes **14b**, and *o*-phenylenediamines **19a** (bottom) [1,30,32,33,34,35].

Dyker et al. investigated the tandem cyclization of substituted 2-ethynylbenzaldehydes **18** with *o*-phenylenediamines **19a** (Figure 15, top pathway) [30]. The preparation of several benzimidazo[2,1-*a*]isoquinolines **1a** in 38–88% yields was reported using nitrobenzene as the solvent and oxidizing agent at elevated temperature (150 °C, 2 d). This significant development offers a metal-catalyst-free preparation for this class of compounds [31].

Dyker’s synthetic design closely relates to the palladium(II) acetate-catalyzed reaction described earlier by Sun and LaVoie [1]. In this prior report, trimethysilyl substituted 2-ethynylbenzaldehyde **18** serves as the starting material in a two-step cyclization/oxidation process via isolated phenylbenzimidazole derivative **20** to form methoxy-nitro-substituted benzimidazo[2,1-*a*]isoquinolines **1a** (R^1^ = OMe, R^2^ = H, R^3^ = NO_2_; 60% overall yield; Figure 15). Though an important contribution, this approach was not studied beyond a reaction that yielded a mixture of two regiomeric 9- and 10-nitro derivatives [1].

Yanada et al. [32] achieved an improved yield (68%) over Dyker et al. (41%) [30] for the synthesis of an unsubstituted benzimidazo[2,1-*a*]isoquinoline via a microwave-assisted protocol using palladium(II) acetate in DMF. This result motivated the further development of a protocol based on *o*-phenylenediamines that combines a one-pot synthesis tandem cyclization and Sonogashira coupling of *o*-bromobenzaldehydes **21**. In this work, a variety of benzimidazo[2,1-*a*]isoquinolines **1a** were produced with 68–83% yields (Figure 15). 2-Ethynylbenzaldehyde **18** forms in situ in the presence of tetrabutylammonium acetate (Bu_4_NOAc), thus eliminating the need to prepare the precursor separately. The palladium(II) acetate catalyst needed for the alkyne coupling process remains in the reaction medium during the cyclization steps. However, it was not reported if the final cyclization would proceed in the absence of catalyst in the experimental conditions used [32]. This protocol is associated with a stoichiometric amount of base, removal of the high-boiling solvent (DMF), and elevated temperature (120 °C). More recently, Verma et al. described an analogous silver-catalyzed protocol in water (65–93% yield; Figure 15) [33].

**Figure 16:**
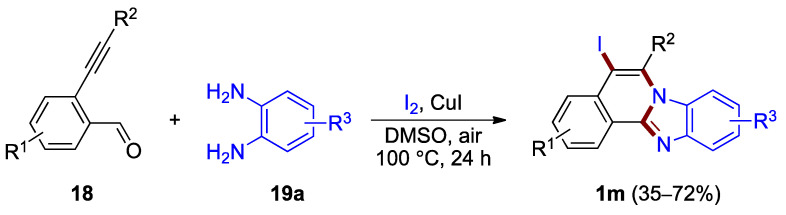
Synthesis of 5-iodobenzimidazo[2,1-*a*]isoquinolines **1m**.

Zhong, Li et al. developed a one-pot method for the synthesis of iodine-functionalized benzimidazo[2,1-*a*]isoquinolines (Figure 16) [36]. A protocol involving a copper(I) iodide catalyst in the presence of iodine produces 5-iodo-substituted benzimidazo[2,1-*a*]isoquinolines **1m** from 2-ethynylbenzaldehydes **18** and *o*-phenylenediamines **19a** in moderate to good yield (35–72%). The analogous bromination reaction using *N*-bromosuccinimide as a halogen source has been reported in one example (45% yield) [36].

**Figure 17:**
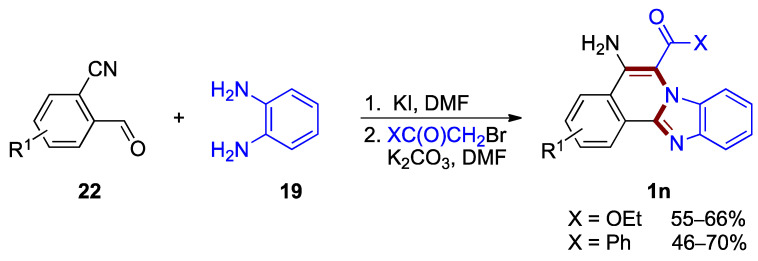
One-pot tandem cyclization leading to 5-amino- and 6-carbonyl functionalized benzimi-dazo[2,1-*a*]isoquinolines **1n**.

Other functionalization of the benzimidazo[2,1-*a*]isoquinoline core can also be achieved by a three-component, one-pot protocol developed for the synthesis of 5-amino- and 6-carbonyl-functionalized benzimidazo[2,1-*a*]isoquinolines **1n** [37]. This transformation, elaborated by Kurth et al., proceeds via a transition-metal free protocol using commercially available *o*-cyanobenzaldehydes **22**, *o*-phenylenediamines **19**, and α-bromoacetate or α-bromoacetophenone (46–70% yields; Figure 17).

**Figure 18:**
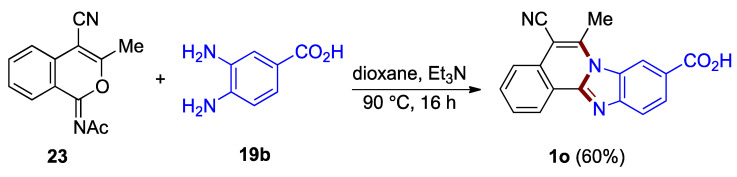
Reaction of acetyliminoisocoumarin **23** and *o*-phenylenediamine **19b**.

5-Cyano-functionalized benzimidazo[2,1-*a*]isoquinoline **1o** has been synthesized from acetyliminoisocoumarin derivative **23** and *o*-phenylenediamine **19b** in the protocol elaborated by Deady et al. (60%; Figure 18) [38]. A related approach utilizes isocoumarin derivatives (30–33%; not illustrated) [3].

The development of catalyst-free protocols that utilize cost-effective conditions and less hazardous solvents has been pursued by Dembinski and Török. Extended studies of the reactivity of alkynes with amines (or related nitrogen nucleophiles [39]) have established that benzodiazepines can be synthesized by the reaction of alkynones and *o*-phenylenediamines in ethanol without a catalyst [40,41]. These experiments confirmed that traditional acid-catalyzed or high-temperature syntheses of heterocycles could readily proceed at mild temperatures without a catalyst to give the intended benzimidazo[2,1-*a*]isoquinolines using substituted *o*-phenylenediamines.

As shown by Török et al., the catalyst-free reaction between a variety of substituted 2-ethynylbenzaldehydes **18** and *o*-phenylenediamines **19a**, at 50 °C, produced the desired products **1a** in 63–92% yields (Figure 15) [34]. The protocol was shown to tolerate strong electron-withdrawing substituents, such as fluorine. The successful installation of a cyclopropyl group at C–6 suggests that the presence of an aryl substituent at the alkyne is not essential to the process. The reaction of 2-ethynylpyridine-3-carbaldehyde demonstrates the utility of the protocol to incorporate additional heteroatoms into the benzimidazo[2,1-*a*]isoquinoline skeleton. Soon after, Koketsu et al. reported also metal-free protocols using the same reagents **18** and **19a** (DMSO, 120 °C; Figure 15), in which produced benzimidazo[2,1-*a*]isoquinolines **1a** (68–84% yields) were subjected to photophysical studies [35].

The reactivity of bifunctional reagents often requires a specific molecular scaffold with pendant functional groups. Regarding the competitive reactivity of nucleophilic aromatic amines versus a carbonyl group or alkyne group, a prior effort suggests that the first step may include the creation of a C–N bond adjacent to the alkyne [40,41]. Alternatively, the first step of the reaction sequence may involve the reaction of the carbonyl group leading to imine formation.

**Figure 19:**
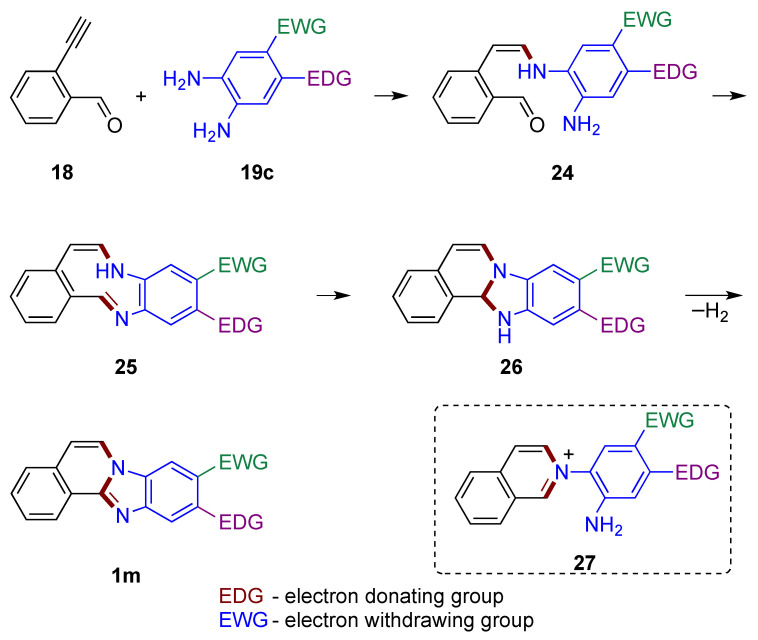
Mechanistic outline illustrating one of the amino groups of *o*-phenylenediamine reacting with the alkyne first.

If alkyne **18** were to first react with *o*-phenylenediamine **19c**, enamine **24** would form adjacent to the aldehyde group (Figure 19). The presence of electron donating group (EDG) and electron withdrawing group (EWG) in non-symmetrical *o*-phenylenediamines affects the nucleophilicity of the amino groups, and in turn determines the regioselectivity of the products. The unreacted amine function of the *o*-phenylenediamine is then available to attack the carbonyl group, forming an imine **25** embedded within the conjugated 9-membered ring system. Nucleophilic attack of the amine on the imine group yields tetracyclic structure **26**, which is further oxidized to the final product **1m** by atmospheric oxygen. The route (not illustrated) leading to the formation of a six-membered ring from attack of the carbonyl group by enamine **24** to give potentially reactive isoquinolinium intermediate **27** should be considered as less likely [33].

**Figure 20:**
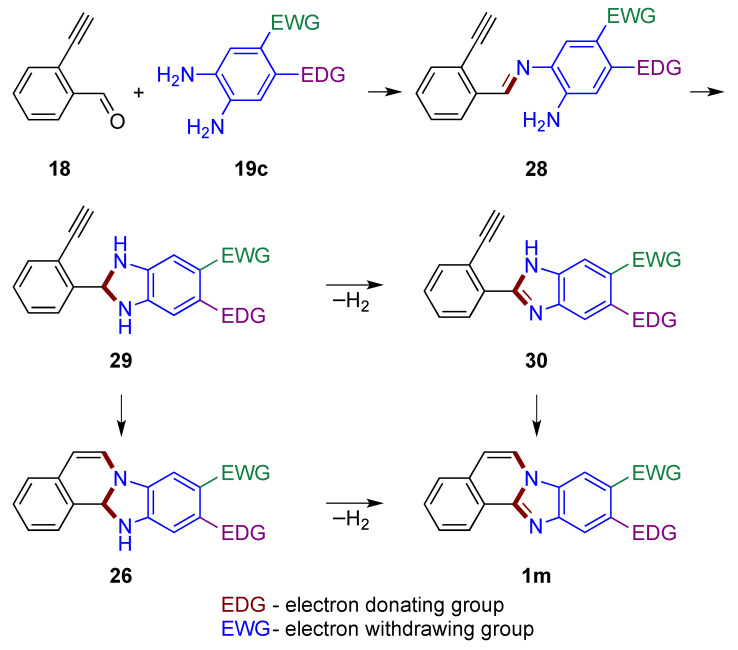
Mechanistic outline for the formation of benzimidazo[2,1-*a*]isoquinolines **1m** with carbonyl group reacting first, illustrating regioselectivity of a reaction using EDG- and EWG-substituted phenylenediamine.

A plausible pathway in which the carbonyl group reacts first is shown in Figure 20. In the first step, the reaction of aldehyde **18** with *o*-phenylenediamine **19c** yields imine **28**. The imine’s carbonyl carbon then connects to the second amino group of the phenylenediamine to form a dihydrobenzimidazole **29**. Oxidation to the benzimidazole **30** (similar to structure **20**, Figure 15) may take place [1,33], which is followed by the attack of the triple bond by the enamine nitrogen atom leading to the final product **1m**. If attack of the triple bond by nitrogen takes place first, leading to the dihydro derivative **26**, subsequent oxidation by atmospheric oxygen yields the final product **1m**. It should be noted that regioselective induction may occur by attack on the alkyne by the more nucleophilic nitrogen in both structures **29** and **30** (possible rotation around a single bond). The EDG and EWG locations illustrated in **19c** impact *o*-phenylenediamine nucleophilic nitrogen atoms in a synergistic manner for both discussed mechanisms (Figure 19 and Figure 20).

**Figure 21:**
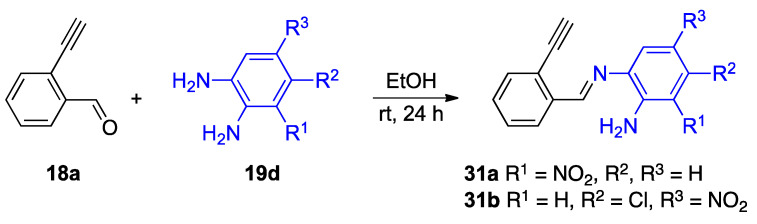
Isolation of mechanistically relevant imine intermediates **31a**,**b**.

The isolation of several intermediates supports the mechanism provided in Figure 20. The reaction of unsubstituted 2-ethnylbenzaldehyde **18a** with selected *o*-phenylenediamines **19d** results in an imine intermediate before a reaction involving the alkyne moiety (Figure 21) [34]. The detection of intermediate **31a**,**b** suggests the reaction at the carbonyl group occurs before the alkyne. Given the electron-withdrawing character of the nitro substituent, the isolation of imines **31a**,**b** also supports the hypothesis that the more electron-rich amino group of the *o*-phenylenediamine reacts soonest [34].

**Figure 22:**
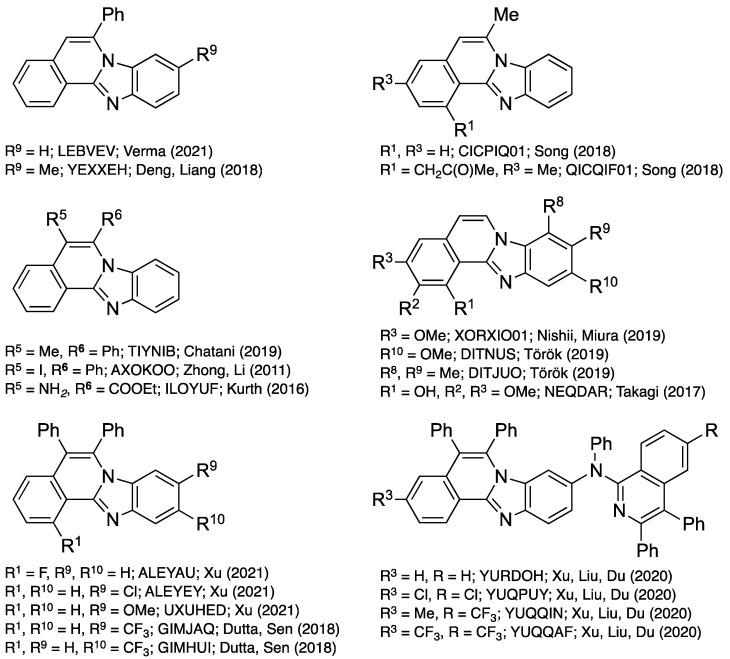
Structures and CSD reference codes of crystallographically characterized derivatives of benzimidazo[2,1-*a*]isoquinolines **1** [10,11,12,13,20,21,27,28,29,33,34,36,37].

The regioselectivity of the reaction of 2-ethnylbenzaldehyde **18** with the 4-methoxy-*o*-phenylenediamine **19d** (EDG = OMe, EWG = H), documented by X-ray crystallography, provides some support for the mechanisms illustrated in Figure 19 and Figure 20. Unambiguous determination of the structure of the major products (CSD ref. code: DITNUS; Figure 22) suggests that the electron donating ability of the methoxy substituent para to the amino group may contribute to the regioselectivity observed. 3,4-Dimethyl-*o*-phenylenediamine was also investigated as a reactant to determine the effect of nucleophilicity on regiochemistry. The regioselectivity observed in the isolated major benzimidazo[2,1-*a*]isoquinoline was confirmed by X-ray crystallography (CSD ref. code: DITJUO; Figure 22). Regioselective induction was observed in other works cited in this review when non-symmetric *o*-phenylenediamines were used as starting materials, demonstrating the formation of regioisomeric mixtures with different degrees of control.

## 6. Crystallographically Characterized Benzimidazo[2,1-*a*]isoquinolines

A search of the CSD shows that several substituted benzimidazo[2,1-*a*]isoquinolines have been crystallographically characterized (Figure 22) [42]. The retrieved 20 structures were sorted by substituent types attached to C–5 and C–6 to review their influence on bond character (length). The substituents’ R numbering in Figure 22 reflects molecules **1** illustrated in Figure 1. The review of bond distance averages is compiled in Table 1, and provides evidence of aromaticity, with pronounced fluctuation of the bond lengths along the derivatives of **1**. Specifically, the C5–C6 bond distance ranges from 1.327(8) to 1.385(2) Å (average 1.35 Å), indicating double bond character, while C4a–C5 and C6–N7 bond distance profiles indicate greater single bond character (1.407(4)–1.464(2) and 1.385(2)–1.418(2)Å, average 1.44 and 1.40 Å, respectively). Presumably, the C5–C6 bond will be the most reactive in the molecule of **1**. The C12a–C12b bond length is notably long with 1.426(2)–1.450(2) Å (average 1.44 Å) bond distances that closely follow C–C bond patterns. The crystal structures also reflect the N12–C12a double bond character (1.307(3)–1.344(3), average 1.32 Å); the distance of single bonds N7–C6, N7–C7a, and N7–C12a was consistent, with averages of 1.40, 1.40, and 1.40 Å, respectively (Table 1).

## 7. Summary and Conclusions

Recently, significant emphasis was placed on the environmental compatibility of the synthesis of heterocycles [43,44], including benzimidazo[2,1-*a*]isoquinolines. Generally, phenyl-substituted benzimidazoles produce benzimidazo[2,1-*a*]isoquinolines in reactions using either ketone or alkyne reagents. Moreover, benzimidamides or ethynylbenzaldehydes can be used to obtain the target compounds through similar reactions with alkynes and diamines, respectively. Widespread interest in the synthesis of benzimidazo[2,1-*a*]isoquinolines has generated a range of effective synthetic protocols, often utilizing alkynes, that differ by the degree of compliance to current environmental and safety standards. This development of synthetic protocols has helped to expand current approaches towards benign and catalyst-free methods [34,35,41,42]. In general, the collected synthetic procedures show broad substituent tolerance and are potentially more amenable to sustainable synthesis than earlier protocols. The green nature of the highlighted method developed in the author’s laboratory is reflected by: (i) catalyst-free protocols; (ii) low temperatures; (iii) renewable solvents; (iv) near quantitative yields; (v) high atom economy; and (vi) readily accessible and cost-effective reagents. Most of the reviewed methods offer vast opportunities for structure enrichment by incorporating, for example, an additional nitrogen heteroatom into ring A and/or D by implementing pyridine-derived starting materials, or for extensions of structures to larger aromatic systems.

## Figures and Tables

**Table 1:** Bond length range and averages [Å] from crystallographic data (for numbering see Figure 1). Bridging internal bonds are highlighted.

Bond	Bond Length Min/Max [Å]		Bond Average [Å]
C1–C2	1.363(4)	1.386(5)	1.38
C2–C3	1.328(3)	1.427(5)	1.40
C3–C4	1.34(1)	1.388(5)	1.37
C4–C4a	1.397(6)	1.441(5)	1.41
C4a–C12b	1.39(2)	1.430(4)	1.41
C4a–C5	1.407(4)	1.464(2)	1.44
C5–C6	1.327(8)	1.385(2)	1.35
C6–N7	1.385(2)	1.418(2)	1.40
N7–C12a	1.377(2)	1.418(4)	1.40
N7–C7a	1.388(2)	1.412(5)	1.40
C7a–C11a	1.392(4)	1.43(1)	1.40
C7a–C8	1.380(4)	1.440(5)	1.40
C8–C9	1.367(4)	1.40(1)	1.38
C9–C10	1.38(1)	1.421(5)	1.40
C10–C11	1.362(4)	1.393(5)	1.38
C11–C11a	1.387(5)	1.411(4)	1.40
C11a–N12	1.364(4)	1.400(4)	1.38
N12–C12a	1.307(3)	1.344(3)	1.32
C12a–C12b	1.426(2)	1.450(2)	1.44
C12b–C1	1.38(2)	1.421(2)	1.40
